# Inferences from the negation of counterfactual and semifactual conditionals

**DOI:** 10.3758/s13421-021-01252-4

**Published:** 2021-11-30

**Authors:** Orlando Espino, Isabel Orenes, Sergio Moreno-Ríos

**Affiliations:** 1 Department of Cognitive, Social and Organizational Psychology, University of La Laguna, Santa Cruz de Tenerife, Spain; 2 Facultad de Psicología, Universidad Nacional de Educación a Distancia (UNED), Madrid, Spain; 3 Facultad de Psicología and CIMCYC, University of Granada, Granada, Spain

**Keywords:** Counterfactual, Semifactual, Mental model, Probabilistic theories, Conditional, Negation

## Abstract

Our goal was to study how people understand the negation of counterfactuals (such as “Antonio *denied*/*said that it is false* that if Messi had played, then Barcelona would have won”) and semifactuals (such as “Antonio *denied that* even if Messi had played, Barcelona would have won”). Previous studies have shown that participants negated basic conditionals using small-scope interpretations by endorsing a new conditional with the negated consequent, but also by making large-scope interpretations, endorsing a conjunction with the negated consequent. Three experiments showed that when participants were asked whether the negation of a counterfactual (Experiments 1 and 2) or semifactual (Experiment 3) conditional was followed by a new conditional, they made a small-scope interpretation, endorsing the same conditional with the negated consequent (e.g., “if/even if Messi had played, Barcelona would *not* have won”). However, they also accepted the conditional with the negated antecedent for semifactuals (e.g., “even if Messi had *not* played, Barcelona would have won”). When participants were asked whether the negation of a counterfactual or semifactual conditional is followed by a conjunction, they endorsed the conjunction with both the negated antecedent and the consequent (e.g., “Messi did *not* play and Barcelona did *not* win”), but again they accepted the conjunction with the negated antecedent only for semifactuals (e.g., “Messi did *not* play and Barcelona did win”). These results have implications for the main theories of reasoning.

## Introduction

Negation has an important functional role in natural human language. All languages have grammatical markers of negation and children from 2 years old are able to properly understand and produce negative statements (Horn, 2001). In spite of this, the literature has shown that negative sentences are harder to understand and reason with compared to their affirmative counterparts (Espino & Byrne, 2012; Khemlani et al., 2012, 2014; Macbeth et al., 2014, 2015, 2017; Moreno-Rios & Byrne, 2018; Orenes & Santamaría, 2014; Wason & Johnson-Laird, 1972). As a result, negation continues to be mysterious and challenging for researchers studying the cognitive processes underlying the comprehension of negation.

Negation is a syntactic operator that takes an argument (e.g., “if Messi had played, then Barcelona would have won”) and reverses its truth value. This means that if an argument is false, then its negation is true (e.g., “it is not the case that if Messi had played, then Barcelona would have won”) and vice versa. We can paraphrase the previous negated counterfactuals as “it is false that if Messi had played, then Barcelona would have won.” An important factor of negation that is under debate is its *scope*, that is, the argument to which the negation applies. In the example above, “it is not the case that if Messi had played, then Barcelona would have won,” negation has a large scope because the scope of the negation is the entire conditional. In contrast, in a sentence such as: “If Messi had played, then Barcelona would *not* have won,” negation has a small scope because the scope of negation is just the consequent of the conditional.

Philosophers have long tried to answer what it is that people understand when they negate a counterfactual (Lewis, 1973; Morato, 2017; Stalnaker, 1968; Williams, 2010), as have psychologists (Pfeifer & Tulkki, 2017), but there is still no consensus on this issue (Nickerson, 2015). Hence, our aim in this paper is to examine how people negate a counterfactual. We will start by reviewing this issue in the literature, presenting the two main theories of reasoning: the probabilistic theories and the mental model theory.

The probabilistic theory of reasoning (Evans & Over, 2004; Oaksford & Chater, 2007, 2013; Over, 2016; Pfeifer & Kleiter, 2010) groups several theories (suppositional theory, Bayesian net, and probability logic) that share the hypothesis that most reasoning is from premises that are uncertain and that this uncertainty, or degrees of belief, in the statements from which we reason can be modelled in probability theory. However, important differences exist between these theories with respect to both their explanatory focus and their core assumptions about the meaning of conditionals (for review see, Manktelow, 2012; Nickerson, 2015). According to probability theories, people understand a conditional “if A then B” by (1) hypothetically adding A to their set of beliefs, (2) making some adjustments so that the resulting set is coherent, then (3) assessing B in the light of this adjusted set of beliefs (Evans, 2007; Evans & Over, 2004; Oaksford & Chater, 2007). This three-step procedure is called the Ramsey test (Ramsey, 1990), and “it lies at the heart of many theories of conditional reasoning in general and counterfactual reasoning in particular” (Lucas & Kemp, 2015, page 3). Some authors in favor of the probabilistic approach in conditional reasoning predict that from the negated conditional “it is not the case that if Messi played, then Barcelona won,” the same conditional with the negated consequent “if Messi played, Barcelona didn’t win” follows (Handley et al., 2006; Pfeifer & Tulkki, 2017). This prediction has been confirmed in several studies (Espino & Byrne, 2012; Handley et al., 2006; Pfeifer & Tulkki, 2017). Likewise, some advocates of the suppositional theory claim that certain conjunctive conclusions, such as “Messi played and Barcelona didn’t win,” should not be endorsed (Handley et al., 2006). The finding that participants do, in fact, judge that the conjunction “Messi played and Barcelona didn’t win” follows from the negation of a conditional has been described as an error in this view. The suppositional theory proposes that it arises because a simulation of “Messi played and Barcelona didn’t win” is consistent with the conditional’s negation (Handley et al., 2006).

According to the probabilistic theories, a counterfactual conditional is understood in the same way as a factual conditional, that is, the probability of a counterfactual conditional at the present time is the same as the probability of the corresponding factual conditional at a previous time (for details, see Over et al., 2007). From this assumption, the negation of counterfactuals should be expected to behave similarly to the negation of conditionals. This was the focus of the work of Pfeifer and Tulkki (2017), and these predictions were corroborated. However, one limitation of this research is that participants were provided with only two options (such as, “if A were the case, then B wouldn’t be the case” and “it is not the case that if A were the case, then B would be the case”) in order to negate a counterfactual. Other options like making conjunctive conclusions (such as “A were the case and B would not be the case”) were excluded in their experiments, given that it is considered an error to infer a conjunction.

An alternative to the probabilistic theories is the mental model theory or model theory (Byrne & Johnson-Laird, 2020; Johnson-Laird & Byrne, 1991, 2002). The model theory proposes that when people understand a factual conditional such as “If Messi played, then Barcelona won,” they simulate the possibilities consistent with its truth. Due to the limitations of working memory, they think only about the events mentioned at the outset (Johnson-Laird et al., 1992):

**Table:** 

Messi played	Barcelona won
…	

The three dots in the diagram indicate that there may be other possibilities. However, people tend to make inferences using just the initial model. But in some circumstances, with enough time, motivation, etc., people flesh out their models to think about some others, for example:

**Table:** 

Messi played	Barcelona won
Messi did not play	Barcelona won
Messi did not play	Barcelona did not win

The conditional rules out the possibility that “Messi played and Barcelona did not win”:

**Table:** 

Messi played	Barcelona did not win

Only when people are asked to think about “it is not the case” with a conditional, do they represent this possibility. Actually, Khemlani et al. (2012) established that the core meaning of the negation of the conditional refers to the complement of the set referred to by that conditional. It follows that a sentential negation contradicts the corresponding affirmation, that is, both cannot be true and false at the same time.

How do people understand and think when they negate a conditional? According to the model theory (Khemlani et al., 2014), people negate a conditional in at least two different ways: they apply the negation to one part of the sentence (small scope negation) or they apply it to the entire sentence (large scope negation). Participants prefer to use the first way if possible. Actually, “the mental model postulates that there is a processing heuristic for negation to be interpreted, where possible, as having a small scope” (Khemlani et al., 2012, page 544). The main reason for this is that the small scope interpretation reduces the number of mental models to be represented. Thus, in this case, people often apply negation in the main clause of the conditional assertion, e.g., “if Messi played then Barcelona did *not* win,” and interpret the negation of a conditional, e.g., “it is not the case that if Messi played, then Barcelona won,” as the small scope negation. However, the possibility “if Messi played then Barcelona did *not* win” does not contradict its corresponding affirmation. Model theory predicts that “the meaning, reference, and knowledge can modulate the core interpretation of negation so that a negative can be merely contrary to its corresponding affirmative assertion” (Khemlani et al., 2012).

The second way in which people negate a conditional is by using the large scope interpretation. In this case, people have to carry out two operations: the enumerative negation process and the comparative process (Khemlani et al., 2014). The first process produces different possibilities by affirming or negating the elements of the initial model possibility. The second process tests whether any of the possibilities are inconsistent with the affirmative conditional. People begin by enumerating the negation of both clauses (Khemlani et al., 2012): *“Messi did not play and Barcelona did not win.”* This possibility is consistent with one of the possibilities of the affirmative conditional, therefore they discard it and continue with the enumerative processing. Next, the possibility *“Messi did not play and Barcelona won”* is enumerated, and again, it is consistent with one of the possibilities of the affirmative conditional, so it is not accepted. Then, the possibility “*Messi played and Barcelona did not win”* is enumerated, but it is not consistent with any of the three possibilities of the affirmative conditional. Finally, they enumerate the possibility “*Messi played and Barcelona won”* and it is consistent with the affirmative conditional, therefore it is rejected. Hence, participants determine that the possibility in which the negation holds is *“Messi played and Barcelona did not win.”* Therefore, the large scope negation of the conditional may lead them to accept the conjunctive possibility “Messi played and Barcelona did not win,” while the small scope negation leads people to accept the conditional “if Messi played then Barcelona did not win.” According to the model theory, the first conclusion is correct while the second one is incorrect. Data from different research shows that people frequently accept both conclusions (Espino & Byrne, 2012; Handley et al., 2006).

However, the enumerative process is not always complete (see also Khemlani et al., 2014), and, therefore, people do not always negate the conditional correctly, given that they do not usually flesh out the implicit models and represent just the initial model “Messi played and Barcelona won” in their understanding of a conditional. Thus, when they enumerate the possibility “Messi did not play and Barcelona did not win,” they realise that it is the one possibility in which the negation holds, because it is not present in the initial model. Some people may stop the enumerative process here, given that it is time-consuming, and conclude that this is the only possibility for the negation of the conditional.

According to model theory, how do people understand and reason with the negation of counterfactuals? Let us start by explaining how people understand and reason with counterfactuals (from a review, Byrne, 2016). The model theory proposes that when people understand a counterfactual conditional, such as “if Messi had played, then Barcelona would have won,” they envisage two possibilities: the conjecture (the imagined possibility), for example, “Messi played and Barcelona won,” and its opposite, the presupposed facts (the factual possibility), for example, “Messi did not play and Barcelona did not win” (e.g., Johnson-Laird & Byrne, 2002):

**Table:** 

*Presupposed Fact*​:	Messi did not play	Barcelona did not win
*Conjectured possibility*​:	Messi played	Barcelona won
…		

The initial representation for a counterfactual is different in two ways from conditionals: there are two initial models instead of one and there are two epistemic labels (Byrne & Johnson-Laird, 2020; Espino & Byrne, 2020). The first model, for example, “Messi did not play and Barcelona did not win,” is a presupposed fact. The second model, for example, “Messi played and Barcelona won,” as in the conditional, is a conjectured possibility with other possibilities represented by the three dots and not explicitly represented (e.g., “Messi did not play and Barcelona won”). People can flesh out their models to think about some of them, for example: “Messi did not play and Barcelona won” and “Messi did not play and Barcelona did not win.” Although counterfactuals have two initial models and conditionals only one, after fleshing out all the models, their full set of consistent possibilities is the same (e.g., Byrne & Johnson-Laird, 2020; Espino & Byrne, 2018). Consequently, the complementary possibility is also the same: for example, “Messi played and Barcelona did *not* win,” which is the core meaning of negation of a counterfactual.

The proposal that from a counterfactual, people think of two possibilities from the outset were corroborated by findings such as that they make the *modus tollens* inference more readily from a counterfactual than from a factual conditional (Byrne & Tasso, 1999) or that they read the conjunction “There were no apples and there were no oranges” more quickly when they are primed by the counterfactual “if there had been apples, then there would have been oranges” compared to the factual conditional “If there are apples, then there are oranges” (Santamaría et al., 2005). According to model theory, the meanings of factual and counterfactual conditionals run in parallel, but their initial mental models differ because they have different epistemic status (Byrne & Johnson-Laird, 2020). Although the epistemic status of some mental models is different between factual conditionals and counterfactuals, the theory does not state how this may influence people’s understanding and reasoning when they negate conditionals or counterfactuals, and consequently the theory of mental models would make the same predictions for a negated counterfactual as for a negated factual conditional.

The main goal of this research is to explore what inferences people endorse when they negate a counterfactual. Unlike Pfeifer and Tulkki’s (2017) research in which only a small range of conclusions (such as, “if A were the case, then B wouldn’t be the case” or “it is not the case that if A were the case, then B would be the case”) was used, in our experiments we present the full set of counterfactual conclusions and conjunctive conclusions. This procedure was previously used by Handley et al. (2006) and Espino and Byrne (2012) to discover how people think when they negate a conditional. This procedure will allow us to find out what type of counterfactual and conjunctive conclusions participants are willing to accept from a negated counterfactual. It is crucial to know how people behave with negated counterfactuals, not only to understand how people reason with counterfactuals, but also in order to distinguish between competing explanations of the cognitive processes underlying counterfactual reasoning and negation.

## Experiment 1: The denied counterfactuals

The goal of this experiment was to explore what inferences people endorse when a counterfactual is negated. In order to negate the counterfactual, we used the word “deny.” This experiment examined the full set of counterfactual and conjunctive conclusions that people judge can follow the negation of a counterfactual, such as “Last year in a conversation about football, Antonio denied that if Messi had played, then Barcelona would have won.” We examined all four possible counterfactual conclusions one by one: “if Messi had played, then Barcelona would have won,” “if Messi had played, then Barcelona wouldn't have won,” “if Messi hadn't played, then Barcelona would have won” and “if Messi hadn't played, then Barcelona wouldn't have won.” We also examined all four possible conjunctive conclusions: “Messi played and Barcelona won,” “Messi played and Barcelona didn't win,” “Messi didn't play and Barcelona won,” and “Messi didn't play and Barcelona didn't win.”

The model theory (Khemlani et al., 2012, 2014) and the probabilistic theory (Pfeifer & Tulkki, 2017) predict that from a negated counterfactual (such as “Antonio denied that if Messi had played, then Barcelona would have won”), participants will infer the same counterfactual with affirmative-antecedent and negated-consequent (such as “if Messi had played, then Barcelona wouldn't have won”). Both theories predict that participants will more frequently choose the conclusion with the counterfactual with affirmative-antecedent and negated-consequent (such as “if Messi had played, then Barcelona wouldn't have won”) than the conclusion with negated-antecedent and affirmative-consequent (such as “if Messi had not played, then Barcelona would have won”) or negated-antecedent and negated-consequent (such as “if Messi had not played, then Barcelona would not have won”) or affirmative-antecedent and affirmative-consequent (such as “if Messi had played, then Barcelona would have won”).

Moreover, according to the model theory (Khemlani et al., 2014), if participants reason correctly from a negated counterfactual, they should more frequently infer a conjunction with affirmative-antecedent and negated-consequent (such as “Messi played and Barcelona did not win”) than a conjunction with negated-antecedent and affirmative-consequent (such as “Messi did not play and Barcelona won”) or negated-antecedent and negated-consequent (such as “Messi did not play and Barcelona did not win”) or affirmative-antecedent and affirmative-consequent (such as “Messi played and Barcelona won”).

### Method

#### Participants

MorePower (6.0.4) Software (Campbell & Thompson, 2012) showed that to achieve 85% power for the effect size obtained in a previous study (ηp^2^ = .085; Espino & Byrne, 2012) with alpha < .05 for 2 × 4 ANOVA, it was necessary to have a sample size of 46 participants in this and the following experiments. The 52 participants in this experiment were undergraduates at the University of La Laguna, Tenerife, Spain and were native speakers of Spanish. There were 45 women and 7 men and their average age was 20 years, range 18–31 years. The experiment received ethical approval from the *Comité de Ética de la Investigación y Bienestar animal* (Committee on Ethics in Research and Animal Welfare, University of La Laguna) prior to its commencement. None of the participants had received instruction in logic nor had they taken part in similar experiments.

#### Design, materials, and procedure

We constructed 24 problems that contained a denied counterfactual, such as “Antonio denied that if Messi had played, then Barcelona would have won” (see Appendix 1. Experimental Materials). Each counterfactual was followed by one of the eight possible conclusions. There were one of the four counterfactual conclusions (e.g., “if Messi had played, then Barcelona would have won,” “if Messi had played, then Barcelona wouldn't have won,” “if Messi hadn't played, then Barcelona would have won,” and “if Messi hadn't played, then Barcelona wouldn't have won”) and four conjunctive conclusions (e.g., “Messi played and Barcelona won,” “Messi played and Barcelona didn't win,” “Messi didn't play and Barcelona won,” and “Messi didn't play and Barcelona didn't win”). Each problem was instantiated in three different conversational topics: football (“Messi played and Barcelona won”), diseases (“the government closed the border and controlled the coronavirus”), and investments (“football clubs invested more money in improving facilities and more fans attended football matches”), that is, 24 problems in total. In each problem the antecedent of the counterfactual was an action (e.g., “Messi played”) and the consequent was a possible result of this action (e.g., “Barcelona won”). So each participant saw each of the three counterfactual conditionals eight times, each time associated with a different conclusion (counterfactual conditional or conjunction). Each conclusion – either counterfactual or conjunction – was the combination of affirmed or denied antecedent and consequent. The participants’ task was to evaluate whether or not a conclusion followed from the denied counterfactual. An example of their task is as follows:

Last year in a conversation about football, Antonio denied that if Messi had played, then Barcelona would have won.

From this statement one can necessarily conclude that:

If Messi had played, then Barcelona would not have won.

YES[ ]NO [ ]

Participants were instructed that their task was to decide whether the proposed conclusion followed from the premise. In order to choose the conclusion, they had to pick YES or NO. The task was presented online using PsyToolkit (Stoet, 2010, 2017) and the problems were presented in a different random order to each participant, with the problems presented one by one. Participants received the following instructions in the first presentation online:

“This task is designed to test your understanding of logical rules. On the following pages you will be presented with a series of problems. In each problem, a rule will be presented, followed by a conclusion. For each problem you must indicate whether or not the conclusion necessarily follows, given the rule that precedes it. If you think that the conclusion follows the rule, you should select the option “yes,” if you think that the conclusion does not follow the rule, you should select the option “no.” A conclusion is necessarily true when the conclusion must follow, given the truth of the rule.”

### Results and discussion

The data for this experiment and the subsequent ones are available online at OSF https://osf.io/3frkg/?view_only=dad4cb01287b4e03bafe5c46c1ca0c60. We carried out a 2 (conclusion connective: counterfactual, conjunction) × 4 (conclusion polarity: A B, A not-B, not-A B, not-A not-B) repeated-measures analysis of variance (ANOVA) on the “yes” responses, using a Greenhouse Geisser correction for the violation of sphericity assumption. There was no main effect of conclusion connective, *F*(1, 51) = .34, *MSE* = .06, *p* = .56, ηp^2^ = .01; a main effect of conclusion polarity, *F*(2.603, 132.773) = 11.51, *MSE* = .16, *p* < .001, ηp^2^ = .18, and the two variables interacted, *F*(2.399, 122.367) = 15.67, *MSE* = .11, *p* < .001, ηp^2^ = .24. The interaction showed that people accepted the counterfactual conclusion “If A, then not-B” more often than the counterfactual “if A, then B” (51% vs. 21%; *F*(1, 51) = 16.65, *MSE* = .15, *p* < .001, ηp^2^ = .25), “if not-A, then B” (51% vs. 26%; *F*(1, 51) = 13.39, *MSE* = .13, *p* = .001, ηp^2^ = .21), and “if not-A, then not-B” (51% vs. 33%; *F*(1, 51) = 5.19, *MSE* = .17, *p* = .027, ηp^2^ = .07). Appendix 2 shows the rest of the comparison. Participants accepted the conjunctive conclusion “not-A and not-B” more often than the conjunctive “A and B” (63% vs. 19%; *F*(1, 51) = 39.76, *MSE* = .13, *p* < .001, ηp^2^ = .44), “A and not- B” (63% vs. 24%; *F*(1, 51) = 30.23, *MSE* = .13, *p* < .001, ηp^2^ = .37) and “not-A and B” (63% vs. 30%; *F*(1, 51) = 22.58, *MSE* = .12, *p* < .001, ηp^2^ = .31). Appendix 2 shows the rest of the comparison (see Table 1).

**Table 1:** Percentages of “yes” responses that people infer from the denied counterfactuals (“X denied that if they had performed action A, then B would have happened”) in Experiment 1

Conclusion polarity:	A B	A not-B	not-A B	not-A not-B
Conclusion connective:				
Counterfactual	21	51	26	33
Conjunction	19	24	30	63

Experiment 1 showed that from the denied counterfactuals, participants mostly infer two conclusions. First, when they have to evaluate whether or not a counterfactual conclusion follows from a denied counterfactual, they more frequently infer a counterfactual conclusion with affirmative-antecedent and negated-consequent (If A, then not-B) than other conclusions (affirmative-antecedent and affirmative-consequent, negated-antecedent and affirmative-consequent, and negated-antecedent and negated-consequent). These results can be predicted by the model theory (Khemlani et al., 2012, 2014) and the probabilistic logic theory (Pfeifer & Tulkki, 2017), although there are marked theoretical differences between these authors when it comes to explaining why people infer a counterfactual with the negated consequent from a negated counterfactual.

Second, when participants have to evaluate whether or not a conjunctive conclusion follows from a denied counterfactual, they more frequently infer a conjunctive conclusion with negated-antecedent and negated-consequent than other conjunctive conclusions (affirmative-antecedent and affirmative-consequent, affirmative-antecedent and negated-consequent, and negated-antecedent and affirmative-consequent). These results are problematic for those theories that claim people use the Ramsey test in order to infer a conclusion (Suppositional theory, and Probabilistic logic). Initially these results could be problematic for model theory if it is assumed that people have reasoned correctly. According to the model theory, if people have reasoned correctly, they should accept the conjunctive conclusion “A and not-B” as correct (Byrne & Johnson-Laird, 2020). However, Khemlani et al. (2012, 2014) proposed that participants tend to start the enumerative process by negating the two clauses (e.g., “Messi did not play and Barcelona did not win”). If this were the case, participants would have discarded this result as the negation of a counterfactual, given that this possibility matches one initial model of the counterfactual, the presupposed fact. Instead, it was the most accepted conclusion. Later, we offer a hypothesis based on a heuristic strategy that explains why people accept the conjunctive conclusion “not-A and not-B” from the negated counterfactual. The hypothesis we offer is compatible with the principles of model theory, but not with probabilistic logic theory.

## Experiment 2: The false counterfactuals

Khemlani et al. (2012) have referenced two important ways of interpreting negation: (1) as denial: to deny preconceptions, and (2) as falsity: to reverse the true value of a sentence (see Khemlani et al., 2012). In the same way, Oversteegen and Schilperoord (2014) mentioned “denial” and “falsity” as two kinds of negations. In Experiment 1 we used the first kind of negation: “Someone denies.” In order to test whether the results were generalized to expressions corresponding to the second type of negation: “Someone said it is false,” we decided to add Experiment 2. So the motivation of this experiment was to replicate the results of Experiment 1, but using the linguistic expression “it is false” instead of the word “deny.”

### Method

#### Participants

The 50 participants in this experiment were undergraduates at the University of La Laguna, Tenerife, Spain and were native speakers of Spanish. There were 42 women and 8 men and their average age was 22 years, range 18–53 years. The experiment received ethical approval from the *Comité de Ética de la Investigación y Bienestar Animal* (Committee on Ethics in Research and Animal Welfare, University of La Laguna) prior to its commencement. None of the participants had received instruction in logic nor had they taken part in similar experiments.

#### Design, materials, and procedure

Design, materials, and procedure were similar to Experiment 1, with the only exception being the replacement of “deny” with “it is false.”

### Results and discussion

We carried out a 2 (conclusion connective: counterfactual and conjunction) × 4 (conclusion polarity: A B, A not-B, not-A B, not-A not-B) repeated-measures analysis of variance (ANOVA) on the “yes” responses, using a Greenhouse Geisser correction for the violation of the sphericity assumption. There was no main effect of conclusion connective, *F*(1, 49) = .49, *MSE* = .05, *p* = .49, ηp^2^ = .01; a main effect of conclusion polarity, *F*(1.630, 79.864) = 15.80, *MSE* = .25, *p* < .001, ηp^2^ = .24, and the two variables interacted, *F*(2.007, 98.364) = 36.46, *MSE* = .10, *p* < .001, ηp^2^ = .43. The interaction showed that people accepted the counterfactual conclusion “If A, then not-B” more often than the counterfactuals “if A, then B” (59% vs. 21%; *F*(1, 49) = 27.77, *MSE* = .14, *p* < .001, ηp^2^ = .36), “if not-A, then B” (59% vs. 23%; *F*(1, 49) = 45.96, *MSE* = .07, *p* < .001, ηp^2^ = .48), and “if not-A, then not-B” (59% vs. 25%; *F*(1, 49) = 23.83, *MSE* = .12, *p* < .001, ηp^2^ = .33). Appendix 2 shows the rest of the comparison. Participants accepted the conjunctive conclusion “not-A and not-B” more often than the conjunctives “A and B” (67% vs. 11%; *F*(1, 49) = 106.47, *MSE* = .08, *p* < .001, ηp^2^ = .69), “A and not-B” (67% vs. 28%; *F*(1, 49) = 21.68, *MSE* = .18, *p* < .001, ηp^2^ = .31), and “not-A and B” (67% vs. 28%; *F*(1, 49) = 18.81, *MSE* = .21, *p* < .001, ηp^2^ = .28). Appendix 2 shows the rest of the comparisons (see Table 2).

**Table 2:** Percentages of “yes” responses that people infer from the false counterfactuals (“X said it is false that if they had performed action A, then B would have happened”) in Experiment 2

Conclusion polarity:	A B	A not-B	not-A B	not-A not-B
Conclusion connective:				
Counterfactual	21	59	23	25
Conjunction	11	28	28	67

Experiment 2 replicates the results obtained in Experiment 1. Although the two expressions could induce different kinds of negation (see Khemlani et al., 2012; Oversteegen & Schilperoord, 2014) (the one used in Experiment 1 related to denying while the one used in Experiment 2 related to falsity), participants’ inferences negating counterfactuals followed the same pattern. Experiment 2 showed that from a false counterfactual, participants mostly infer two conclusions. First, when they have to evaluate whether or not a counterfactual conclusion follows from a false counterfactual, they more frequently infer a counterfactual conclusion with affirmative-antecedent and negated-consequent (“If A, then not-B”) than other counterfactual conclusions (affirmative-antecedent and affirmative-consequent, affirmative-antecedent and negated-consequent, and negated-antecedent and negated-consequent). As in Experiment 1, these results can be predicted by the model theory (Khemlani et al., 2012; Stalnaker, 1968) and the probabilistic logic theory (Pfeifer & Tulkki, 2017).

Second, when participants have to evaluate whether or not a conjunctive conclusion follows from a false counterfactual, they more frequently infer a conjunctive conclusion with negated-antecedent and negated-consequent (such as “not-A and not-B”) than other conjunctive conclusions (affirmative-antecedent and affirmative-consequent, affirmative-antecedent and negated-consequent, and negated-antecedent and affirmative-consequent). As in Experiment 1, these results are not predicted by theories that claim people use the Ramsey test in order to infer a conclusion (Suppositional theory and Probabilistic logic). Moreover, these results do not fit the predictions of the model theory either, since if people had reasoned correctly with false counterfactuals, they would have chosen the conjunctive conclusion with affirmative-antecedent and negated-consequent (such as, “A and not-B”).

How then can we explain these results? Counterfactual conditionals and basic conditionals tell us about the conjectured possibilities, but in addition, counterfactual conditionals tell us about a presupposed fact. Therefore, a counterfactual is similar to a basic conditional but with two epistemic realities instead of just one. The interpretation of the negation could lie in thinking that only one of the two realities is possible. It can be made by discarding the conjectured possibilities and accepting the presupposed fact (“not-A and not-B”) as the negation of the counterfactuals. But there is another way: discarding the presupposed fact and negating the conditional as a basic conditional (see Table 3). If people choose to proceed the first way, they reject the conjecture and accept the fact. Note that it is more difficult for the fact to mutate, since there are no alternatives, and therefore people conclude with the fact “not-A and not-B” as the negation of the counterfactual. For example, when people read the negated counterfactual “it is not the case that if Messi had played, Barcelona would have won” they assume that the conjectured possibilities are false, discarding all the possibilities (such as “Messi played and Barcelona won”) and accepting the factual possibility: “Messi did not play and Barcelona did not win.”

**Table 3:** Strategies people use in negating counterfactual and semifactual conditionals

	Counterfactual “It is not the case that if Messi had played, Barcelona would have won”	Semifactual “It is not the case that even if Messi had played, Barcelona would have won”
Initial representation (without denial)	Fact: Not-A Not-B Conjecture: A B …	Fact: Not-A B Conjecture: A B …
Complete representation (without denial)	Fact: Not-A Not-B Conjecture: A B Not-A Not-B Not-A B	Fact: Not-A B Conjecture: A B Not-A Not-B Not-A B
Deny by discarding the conjecture and accepting the fact	Not-A Not-B “Messi did not play and Barcelona did not win”	Not-A B “Messi did not play and Barcelona won”
Deny by discarding the fact and enumerating cases to test with representations	With initial representations: Not-A Not-B “Messi did not play and Barcelona did not win” With partial fleshing out: Not-A B With complete representation: A Not-B	With initial representations: Not-A Not-B “Messi did not play and Barcelona did not win” With partial fleshing out: Not-A B With complete representation: A Not-B

If people choose the second way to proceed, the negation of a counterfactual is equivalent to the negation of a basic conditional “it is not the case that if Messi played, Barcelona won,” given that the factual possibility is discarded. Participants operate only with the conjectured possibility (“Messi played and Barcelona won”) and they know that there are other implicit alternatives (implicit models). Thus, denying a counterfactual leads to the same result as denying a conditional: participants could carry out an incomplete enumeration process and accept the “not-A and not-B” case. The enumerative negation process starts with the negation of each clause of the negated conditional: for example, “Messi did not play and Barcelona did not win.” This possibility is not consistent with the conjecture (“Messi played and Barcelona won”) and therefore many participants will conclude that this possibility is the negation of the conditional and stop the enumerative process. Only some participants will notice that it is indeed consistent, if they flesh out the implicit models. In this case, they will continue with the enumerative process, negating one of the two clauses. In any case, if they continue the enumerative process, they will apply the negation to only one of the two clauses. The possibility “Messi played and Barcelona did not win” is inconsistent with the conjectured models, and therefore, participants could conclude that this case is consistent with the negation of the conditional. In both cases the most frequent expected response is the same: “not-A and not-B.” Therefore, using counterfactuals, it is not possible to distinguish between the two explanations (see Table 3). The reason is that the factual possibility and the possibility generated from the partial enumerative processing are the same. This does not happen with semifactual conditionals, which have a different factual possibility. In Experiment 3, we will replace the counterfactual conditional with the semifactual conditional.

## Experiment 3: The denied semifactuals

According to the model theory, when people understand a semifactual conditional such as “even if Messi had played, Barcelona would have won,” they envisage dual possibilities, that is, they construct a model corresponding to the conjectured possibility, “Messi played and Barcelona won,” and one corresponding to the factual possibility (the presupposed fact), “Messi did not play and Barcelona won” (Byrne, 2016):

**Table:** 

*Presupposed Fact*:	Messi did not play	Barcelona won
*Conjectured possibility*:	Messi played	Barcelona won
…		

Experiment 3 will be conducted to test the two strategies based on the epistemic status derived from the mental model theory. They involve (1) discarding the presupposed fact and proceeding with the conjecture and implicit possibilities as with basic conditionals. In this case, given that the only model that differs between counterfactuals and semifactuals is the discarded model, the negation of semifactuals will lead to the same results as the negation of counterfactual conditionals. Therefore, as in Experiments 1 and 2, the “not-A and not-B” conjunction will be the one most frequently chosen (e.g., “Messi did not play and Barcelona did not win”). However, if participants use the second strategy, a different result is predicted: (2) the most mutable possibility (the conjecture) is discarded and participants conclude with the factual possibility “not-A and B” (e.g., “Messi did not play and Barcelona won”). As predicted by the mental model theory and probabilistic theories, participants will apply the small scope strategy when possible, negating the main clause. Thus, they would prefer to conclude that the negation of the semifactual, i.e., “even if Messi had played, Barcelona would have won” is “even if Messi had played, Barcelona would not have won.”

### Method

#### Participants

The 83 participants in this experiment were undergraduates at the University of Granada, Spain and were native speakers of Spanish. There were 66 women and 17 men and their average age was 19 years, range 17–50 years. The experiment received ethical approval from the *Comité de Ética en Investigación Humana* (Committee on Ethics in Human Research, Universidad of Granada, R.N. 1068/CEIH/2020). None of the participants had received instruction in logic nor had they taken part in similar experiments.

#### Design, materials, and procedure

Design, materials, and procedure were similar to Experiment 1, with the only exception being that counterfactual expressions were replaced by semifactuals.

### Results and discussion

We carried out a 2 (conclusion connective: semifactual and conjunction) × 4 (conclusion polarity: A B, A not-B, not-A B, not-A not-B) repeated-measures analysis of variance (ANOVA) on the “yes” responses, using a Greenhouse Geisser correction for the violation of the sphericity assumption. There was a main effect of conclusion connective, *F*(1, 82) = 41.00, *MSE* = .04, *p* < .001, ηp^2^ = .33; a main effect of conclusion polarity, *F*(2.281, 170.660) = 9.99, *MSE* = .23, *p* < .001, ηp^2^ = .11, and the two variables interacted, *F*(2.908, 203.59) = 9.83, *MSE* = .09, *p* < .001, ηp^2^ = .12. The interaction showed that people accepted the conclusion “even if A, not-B” more often than the conclusions “even if A, B” (58% vs. 33%; *F*(1, 82) = 14.02, *MSE* = .20, *p* < .001, ηp^2^ = .15), and “even if not-A, not-B” (58% vs. 27%; *F*(1, 82) = 41.38, *MSE* = .10, *p* < .001, ηp^2^ = .34). Also they accepted the conclusion “even if not-A, B” more often than the conclusions “even if A, B” (52% vs. 33%; *F*(1, 82) = 22.71, *MSE* = .07, *p* < .001, ηp^2^ = .22), and “even if not-A, not-B” (52% vs. 27%; *F*(1, 82) = 19.33, *MSE* = .13, *p* < .001, ηp^2^ = .19). They accepted the conclusion “even if A, not-B” as frequently as “even if not-A, B” (58% vs. 52%; *F*(1, 82) = .85, *MSE* = .20, *p* = .36, ηp^2^ = .01) and they accepted the conclusion “even if A, B” as frequently as “even if not-A, not-B” (33% vs. 27%; *F*(1, 82) = .99, *MSE* = .11, *p* = .32, ηp^2^ = .01).

As we predicted, they accepted the conjunctive conclusion “not-A and not-B” as frequently as the conclusion “not-A and B” (45% vs. 46%; *F*(1, 82) = .07, *MSE* = .16, *p* = .80, ηp^2^ < .001) but more frequently than the conclusion “A and B” (45% vs. 16%; *F*(1, 82) = 30.84, *MSE* = .11, *p* < .001, ηp^2^ = .27), and “A and not-B” (45% vs. 22%; *F*(1, 82) = 19.35, *MSE* = .11, *p* < .001, ηp^2^ = .19). Also, they accepted the conclusion “not-A and B” more frequently than the conclusion “A and B” (46% vs. 16%; *F*(1, 82) = 41.61, *MSE* = .09, *p* < .001, ηp^2^ = .34), and “A and not-B” (46% vs. 22%; *F*(1, 82) = 25.50, *MSE* = .10, *p* < .001, ηp^2^ = .24). Finally, they accepted the conclusion “A and B” as frequently as the conclusion “A and not-B” (16% vs. 22%; *F*(1, 82) = 1.76, *MSE* = .06, *p* = .19, ηp^2^ = .02) (see Table 4).

**Table 4:** Percentages of “yes” responses that people infer from the denied semifactuals (“X denied that even if they had performed action A, B would have happened”) in Experiment 3

Conclusion polarity:	A B	A not-B	not-A B	not-A not-B
Conclusion connective:				
Semifactual	33	58	52	27
Conjunction	17	22	45	46

Experiment 3 showed that from a denied semifactual, participants infer the semifactual with affirmative-antecedent and negated-consequent (“even if A, not-B”) as frequently as the semifactual with negated-antecedent and affirmative-consequent (“even if not-A, B”). They infer these two conclusions more frequently than the other two conclusions (“even if A, B” and “even if not-A, not-B”). Therefore, this experiment showed that participants apply the small-scope negation strategy to infer that a semifactual conclusion follows from a denied semifactual. In contrast to the denied counterfactual (Experiments 1 and 2), for a denied semifactual they apply the small-scope negation strategy over the main clause (“even if A, not-B”) and over the subordinate clause (“even if not-A, B”).

Participants infer the conjunctive conclusion with negated-antecedent and negated-consequent (“not-A and not-B”) as frequently as the conjunctive conclusion with negated-antecedent and affirmative-consequent (“not-A and B”) from a denied semifactual. Also, they infer these two conjunctive conclusions more frequently than the conjunctive conclusion with affirmative-antecedent and affirmative-consequent (“A and B”) and the conjunctive conclusion with affirmative-antecedent and negated-consequent (“A and not-B”). As in Experiments 1 and 2 with counterfactuals, participants conclude “not A and not B,” but unlike in those experiments, participants also conclude the “not-A and B.” Therefore, the result shows that denying semifactual conditionals is done in a different way from denying conditionals or counterfactuals. The result is also consistent with the proposal that participants deny semifactuals (and counterfactuals) by discarding one of the two possibilities of the initial model: sometimes they discard the conjecture and accept the factual possibility “not-A and B” and at other times they discard the factual possibility and operate over the mutable model as in conditionals, concluding by the enumerative process, “not A and not B.” Consequently, we can conclude that when participants have to infer conjunctive conclusions from a denied counterfactual, they use a heuristic strategy based on the epistemic role of possibilities.

Finally, Experiment 3 has shown that there is more than one way of denying counterfactuals and has allowed us to rule out possible simple explanations. For example, the results in Experiments 1 and 2 were consistent with denying counterfactuals by selecting the presupposed model and discarding the conjectured model. However, the results were also consistent with the opposite: discarding the presupposed model and selecting the conjectured one, and therefore, operating as with a basic conditional. Experiment 3 has shown that one strategy alone cannot explain how people deny conditionals, as the results were consistent with two: people selected two conjunctive conclusions: the factual possibility (“not-A and B”) and the conjecture possibility with antecedent and consequent with reversed polarity (“not-A and not-B”).

## General discussion

Experiments 1 and 2 show that participants mostly infer two types of conclusion from a denied/false counterfactual. When they evaluate whether or not a counterfactual conclusion follows from the denied/false counterfactual “X denied/X said it is false if A, then B,” they infer the counterfactual “if A, then not-B” more frequently than other counterfactual conclusions (“if A, then B,” “if not-A, then B,” and “if not-A, then not-B”). These results suggest that people use the small-scope negation strategy to infer what follows from a denied/false counterfactual. The use of the small-scope negation strategy is predicted by both the model theory (Khemlani et al., 2012, 2014) and the probabilistic theory (Pfeifer & Tulkki, 2017). However, these theories offer different hypotheses in order to explain this result. For some authors, this conclusion is correct or valid (Pfeifer & Tulkki, 2017; Stalnaker, 1968), while for other authors, it is an incorrect conclusion (Khemlani et al., 2012, 2014; Lewis, 1973). These results replicate those obtained by other authors with negated counterfactuals (Pfeifer & Tulkki, 2017) or negated conditionals (Espino & Byrne, 2012; Handley et al., 2006; Khemlani et al., 2014). However, they do not allow us to determine whether people are reasoning correctly (Handley et al., 2006; Pfeifer & Tulkki, 2017; Stalnaker, 1968) or wrongly (Espino & Byrne, 2012; Khemlani et al., 2014; Lewis, 1973). These results could not be explained either by Egré and Politzer (2013) or Politzer et al. (2020). According to these authors, the negation of “if A then B” is equivalent to the weak negation “if A, possibly not B”. They assume that this modal negation is the baseline negation for all indicative conditionals, but they also claim that the other two possible strong conclusions (such as, “if A, then not B’ and “A and not B”) could possibly occur, depending on the level of belief in the affirmative conditional sentence and its antecedent. Egré and Politzer (2013) predicted that people would tend to use the strong conclusion “if A, then not B” only if they were sufficiently doubtful of the conditional, and they would tend to use the strong conclusion “A and not B” over both conditional forms (“if A, it might be that not B”, or “if A, then not B”) only if they were sufficiently confident in the truth of the antecedent. The experiments we present here can neither confirm nor rule out the main predictions of Egré and Politzer (2013) and Politzer et al. (2020) for several reasons. Firstly, in our experiment we did not manipulate the probabilities of the conditional and the antecedent, which could determine whether or not participants favored a strong or weak conclusion. Secondly, the predictions of these authors refer to negated conditionals but not to negated counterfactuals. Thirdly, even if we assume that denial counterfactuals behave the same way as denial conditionals, the fact that people accept the conjunctive conclusion “not A and not B” more frequently than other conjunctive conclusions (Experiment 1 and Experiment 2) is difficult for Egré and Politzer (2013) and Politzer et al. (2020) to explain.

Similar results to those of Experiments 1 and 2 were obtained in Experiment 3, which used denied semifactuals. When participants have to evaluate whether or not a semifactual conclusion follows from a denied semifactual “X denied even if A, B,” they infer the semifactual conclusion “even if A, not-B” and “even if not-A, B” more frequently than other semifactual conclusions (“even if A, B” and “even if not-A, not-B”). These results suggest that people use the small-scope negation strategy in order to infer what follows from a denied semifactual. However, unlike in Experiments 1 and 2, participants apply small-scope negation strategy over the main clause (such as “even if A, not-B”) and over the subordinate clause (such as “even if not-A, B”). One possible explanation as to why they used the small-scope negation strategy over the main and subordinate clause is that they interpreted the semifactual as a biconditional (such as “X denied even if A, B” and “X denied even if not-A, not-B”). In the first case (“X denied even if A, B”), if participants apply the small-scope negation strategy over the main clause, they can infer the semifactual conclusion “even if A, not-B.” In the second case (“X denied even if not-A, not-B”), if participants apply the small-scope negation strategy over the subordinate clause, they can infer the semifactual conclusion “even if not-A, B.” Espino and Byrne (2012) found the same pattern with negated conditionals. Another possible explanation is based on the fact that “even if A, then B” (“aunque” in Spanish) is a concessive-conditional in the subjunctive mood (Flamenco, 1999; König, 1986; Montolío, 1999). To apply the small scope strategy, some participants might be trying to preserve the concessive character of the sentence, which requires them to maintain the polarity in the main clause (B must happen in any case). The only way to do this is by negating the subordinate clause (Even if not A, then B).

Experiments 1 and 2 showed that when they have to evaluate whether or not a conjunctive conclusion follows from a denied/false counterfactual (“X denied/X said it is false if A, then B”), they infer the conjunctive conclusion “not-A and not-B” more frequently than the other conjunctive conclusions (“A and B,” “A and not-B,” and “not-A and B”). These results are problematic for those theories that claim people use the Ramsey test in order to infer a conclusion (Suppositional theory and Probabilistic logic). The Ramsey test implies that when people make inferences, they do not think of false antecedents (such as “not-A”), they only think of true antecedents (such as “A”). These results are also problematic for the model theory if we assume that participants have been thinking correctly (Byrne & Johnson-Laird, 2020). According to the model theory, if the participants have reasoned correctly, they should judge the conjunctive conclusion “A and not-B” as correct in the denied/false counterfactual and semifactual conditionals. The theory can explain this result with conditionals based on incomplete enumerative processing (Khemlani et al., 2014). Participants start negating the two clauses and construct the “not-A and not-B” case. This possibility is not represented as an initial model in the core meaning of a basic conditional. Therefore, some participants could accept this possibility as the negation of the conditional and stop the process without fleshing out the implicit possibilities. However, this cannot be explained with regard to counterfactuals, because it is represented in the set of initial mental models: people represent the conjecture and the presupposed fact when they understand counterfactuals.

In order to explain why participants select the conclusion with negated antecedent and negated consequent (i.e., “not-A and not-B”) from a negated counterfactual and semifactual, we propose that participants were using two possible heuristic strategies based on the epistemic role of possibilities. On the one hand, participants could be discarding the presupposed fact from the initial representation and proceeding with the conjecture. This might account for their acceptance of the negated antecedent and consequent (i.e., “not-A and not-B”) after an incomplete enumerative process without fleshing out the implicit model, as predicted for conditionals. Thus, after reading that it is false that “if Messi had played, Barcelona would have won” a first group of people could direct their attention to what really happened (Messi did not play and Barcelona did not win), these people could then discard the presupposed fact and proceed to negate the conjectured possibilities (the conjectured cases: Messi played and Barcelona won; Messi did not play and Barcelona won). Alternatively, they could be discarding the conjecture (e.g., Messi played and Barcelona won) and accepting the presupposed facts (e.g., Messi did not play and Barcelona did not win). The result is exactly the same: the acceptance of “not-A and not-B”. In both cases, the mental model theory could easily accommodate this result.

Why do people operate on the conjecture in the first case and in the second case just accept the presupposed facts? If they discard the presupposed facts, they are opting for a set of possibilities, not for a fact: there is a mutable conjectured possibility with other alternatives (signalled by the implicit model). However, if they discard the conjecture, they are opting for a fact. Facts are not mutable. Unfortunately, our research does not allow us to test what makes some people direct their attention to one or the other model or to use a different strategy. In the future, these strategies could be tested using comprehension tasks, obtaining reading times and eye-movement technics and manipulating the focus of the participants in the story to attend to the conjectured or presuppositional cases.

When participants operate with the conjectured possibility (discarding the factual possibility), they look for different ways to negate the clauses in the conditional (see Khemlani et al., 2012, 2014) starting with the negation of the two clauses, for example, in the negated counterfactual and semifactual “X denied/said it is false if/even if A, then B”, enumerating the “not-A and not-B” case. This possibility is not consistent with their representations, as the presupposed facts have been discarded, and therefore it is a possible solution, putting a stop to the enumerative process. Results in the three experiments show that this conclusion is more frequent than even the correct conclusion “A and not B.” Also, notice that unless the presupposed fact is discarded, this conclusion is not predicted with counterfactuals, given that the “not-A and not-B” is already represented in the initial models of counterfactuals. On the other hand, when participants discard the conjecture, predictions for counterfactuals and semifactual conditionals are different: again “not-A and not-B” is the predicted response only for counterfactuals. Results in Experiments 1 and 2 show that the frequency for that option is higher than any other. The factual possibility for semifactual conditionals is “not-A and B,” therefore, the two strategies lead to different conclusions. Accordingly, results in Experiment 3 show that the two predicted options are the most frequent conclusions with no difference between them, and in both cases, they are more frequent than the correct conclusion.

The main contributions of this research are: (1) systematic research has been carried out on how people infer whether or not a counterfactual/semifactual conclusion or conjunctive conclusion follows from denied/false counterfactuals or semifactuals; (2) how people reason with denied semifactuals has been studied for the first time; (3) two heuristic strategies have been proposed for how people evaluate whether or not a conjunctive conclusion follows from a denied/false counterfactual or from a denied semifactual; and, finally, (4) the data from this research showed that the nature of the conclusion to be evaluated determines how people think. When participants have to evaluate whether or not a counterfactual/semifactual conclusion (such as, “if A, then not-B”) follows from a denied/false counterfactual (such as “X denied if A, then B” or “X said it is false that if A, then B”) or a denied semifactual (such as “X denied even if A, B”), they apply the small-scope negation strategy. On the other hand, when participants have to evaluate whether or not a conjunctive conclusion (such as “A and B”) follows from a denied/false counterfactual or a denied semifactual, they apply two heuristic strategies based on the epistemic role of the possibilities.

In general, the data obtained in this research can be explained according to the main principles (truth, parsimony, dual representation, etc.) of the model theory, as we have shown. However, the data relative to evaluating whether or not a conjunctive conclusion follows from a denied counterfactual or semifactual are problematic for those theories that claim people use the Ramsey test to infer a conclusion (Handley et al., 2006; Pfeifer & Tulkki, 2017). The main problem for probabilistic theories is in assuming that people do not think with false antecedents when they have to evaluate whether a conclusion follows from a counterfactual or semi-factual premise. Our experiments showed the opposite.

## Appendix 1

### Experimental materials

#### Experiments 1 and 2 (in parenthesis)

Last year, in a conversation about football, Antonio denied (said it is false) that if Messi had played, Barcelona would have won - translated from Spanish “El año pasado en una conversación sobre fútbol, Antonio negó (comentó que es falso) que si Messi hubiera jugado, el Barcelona habría ganado.”

Last month in a radio interview, the vice-president of Madrid's government denied (said that it is false) that if the government had closed the border, it would have controlled the coronavirus - translated from Spanish “El mes pasado en una entrevista en la radio, el vicepresidente de gobierno de Madrid negó (comentó que es falso) que si el gobierno hubiera cerrado la frontera, habría controlado el coronavirus.”

In a television programme, one of the interviewees denied (said that it is false) that if the soccer clubs had invested more money in improving facilities, more fans would have attended the soccer matches - translated from Spanish “En un programa de televisión, uno de los entrevistados negó (comentó que es falso) que si los clubs de fútbol hubiesen invertido más dinero en mejorar las instalaciones, más aficionados habrían asistido a los partidos de fútbol.”

#### Experiment 3

Last year in a conversation about football, Antonio denied that even if Messi had played, Barcelona would have won - translated from Spanish “El año pasado en una conversación sobre fútbol, Antonio negó que aunque Messi hubiera jugado, el Barcelona habría ganado.”

Last month in a radio interview, the vice-president of Madrid's government denied that even if the government had closed the border, it would have controlled the coronavirus - translated from Spanish “El mes pasado en una entrevista en la radio, el vicepresidente de gobierno de Madrid negó que aunque el gobierno hubiera cerrado la frontera, habría controlado el coronavirus.”

In a television programme, one of the interviewees denied that even if the soccer clubs had invested more money in improving facilities, more fans would have attended the soccer matches - translated from Spanish “En un programa de televisión, uno de los entrevistados negó que aunque los clubs de fútbol hubiesen invertido más dinero en mejorar las instalaciones, más aficionados habrían asistido a los partidos de fútbol.”

## Appendix 2

### Additional statistical analyses

#### Experiment 1

Counterfactual conclusions:

“if A, B” vs. “if not-A, B”, 21% vs. 26%; *F* < 1

“if A, B” vs. “if not-A, not-B”: 21% vs. 33%; *F*(1, 51) = 4.42, *MSE* = .09, *p* = .04, ηp​^2​^ = .082

“if not-A, B” vs. “if not-A, not-B”: 26% vs. 33%; *F*(1, 51) = 1.07, *MSE* = .12, *p* = .31, ηp^2​^​ = .02

Conjunctive conclusions:

“A & B” vs. “A & not-B”: 19% vs. 24%; *F*(1, 51) = 1.08, *MSE* = .08, *p* = .30, ηp​^2​^ = .022

“A & B” vs. “not-A & B”: 19% vs. 30%; *F*(1, 51) = 4.09, *MSE* = .09, *p* = .045, ηp​^​2​^ = .072

“A & not-B” vs. “not-A & B”: 24% vs. 30%; *F*(1, 51) = 1.48, *MSE* = .06, *p* = .23, ηp​^​2​^ = .03

#### Experiment 2

Counterfactual conclusions:

“if A, B” vs. “if not-A, B”, 21% vs. 23%; *F* < 1

“if A, B” vs. “if not-A, not-B”: 21% vs. 25%; *F*(1, 49)= 1.71, *MSE* = .03, *p* = .20, ηp​^2​^ = .03

“if not-A, B” vs. “if not-A, not-B”: 23% vs. 25%; *F* < 1

Conjunctive conclusions:

“A & B” vs. “A & not-B”: 11% vs. 28%; *F*(1, 49) = 12.62, *MSE* = .06, *p* = .001, ηp​^2​^ = .21

“A & B” vs. “not-A & B”: 11% vs. 28%; *F*(1, 49) = 9.40, *MSE* = .08, *p* = .004, ηp^2​^ = .16

“A & not-B” vs. “not-A & B”: 28% vs. 28%; *F* < 1.
